# STARTing to bridge the evidence gap: management of cancer-associated thrombosis with concomitant thrombocytopenia

**DOI:** 10.1016/j.rpth.2025.103165

**Published:** 2025-09-01

**Authors:** Vasiliki Xirou, Rushad Patell

**Affiliations:** 1 Department of Medicine, Mount Auburn Hospital, Harvard Medical School, Cambridge, Massachusetts, USA; 2 Division of Hematology and Hematologic Malignancies, Department of Medicine, Beth Israel Deaconess Medical Center, Harvard Medical School, Boston, Massachusetts, USA

Venous thromboembolism (VTE) is a common and serious complication in patients with cancer, and represents a frequent indication for anticoagulation [[1], [2], [3]]. Patients with cancer have an increased risk of major bleeding, including bleeding in critical sites such as intracranial hemorrhage [4]. Thrombocytopenia is also frequently associated with malignancy, resulting from either the malignancy itself (in hematologic cancers) or cytotoxic cancer-directed therapies [5]. The coexistence of a hypercoagulable state and low platelet counts is particularly prevalent, affecting up to 20% of patients with solid tumors and nearly 50% of those with hematologic malignancies [6]. This intersection creates a complex clinical scenario, as anticoagulation becomes both necessary and hazardous. Data suggest that even mild thrombocytopenia in the setting of anticoagulation significantly increases the risk of bleeding; however, it is not protective against thrombosis [7,8]. Thus, managing cancer-associated thrombosis (CAT) in the setting of thrombocytopenia requires a careful balance between preventing recurrent VTE and minimizing the risk of bleeding.

Data on the optimal strategy to manage this clinically challenging yet frequent issue are limited. A 2018 systematic review included 2 retrospective studies with a total of 121 patients with CAT and platelet counts below 50 × 10^9^/L who received either dose-modified anticoagulation or full-dose anticoagulation with platelet transfusion [9]. Regardless of their treatment strategy, 27% of those anticoagulated experienced recurrent VTE, and 13% had major bleeding. Due to study heterogeneity, meta-analysis was not feasible, and no single strategy was favored. A more recent systematic review and meta-analysis included 707 adult patients with CAT and platelet counts below 100 × 10^9^/L [10]. Three treatment strategies were identified in this study: full-dose anticoagulation, modified-dose anticoagulation, and no anticoagulation. Low-molecular-weight heparin (LMWH) was the most commonly used agent, and 90% of patients had hematologic malignancies. The analysis did not demonstrate statistically significant differences in bleeding or recurrent VTE across treatment approaches; however, event rates were high across all arms. Notably, the included studies were all at serious risk of bias.

Only 2 multicenter prospective studies have compared anticoagulation strategies specifically in patients with CAT and thrombocytopenia. The Thrombocytopenia Related Outcomes with Venous thromboEmbolism (TROVE) study included 121 patients, 70% of whom had hematologic malignancies, with CAT diagnosed within 7 days and a platelet count below <100 × 10^9^/L [11]. Anticoagulation strategies were categorized as full-dose with platelet transfusion support, modified-dose (including unfractionated heparin, enoxaparin, apixaban, or rivaroxaban) alone, or no anticoagulation. The study found that modified-dose anticoagulation was associated with a lower rate of major hemorrhage, and no recurrent VTE events were observed following its initiation. The Cancer Associated Venous Thrombosis and Thrombocytopenia (CAVEaT) study focused exclusively on patients with hematologic malignancies, including 105 individuals with proximal deep vein thrombosis (DVT) or pulmonary embolism (PE) and thrombocytopenia, defined as platelet counts < 50 × 10^9^/L [12]. Patients received full-dose LMWH or unfractionated heparin, reduced-dose LMWH, direct oral anticoagulants (DOACs), or no anticoagulation, with platelet transfusion thresholds <40 or ≤30 × 10^9^/L across treatment arms. Interestingly, CAVEaT reported numerically lower rates of both bleeding and thrombotic events with full-dose anticoagulation, in contrast to the findings of TROVE. These conflicting findings highlight the limitations of currently available prospective studies, which are primarily constrained by nonrandomized treatment allocation and imbalances in baseline characteristics, including the depth and duration of thrombocytopenia, underlying malignancy, and anticoagulation strategies used.

Given the limited data, expert consensus has informed current management guidelines for CAT in the setting of thrombocytopenia. Recommendations, including those of the International Society on Thrombosis and Haemostasis, suggest that patients with CAT and platelet counts ≥ 50,000/μL should receive full-dose therapeutic anticoagulation without platelet transfusion. For those with platelet counts < 50,000/μL and a high risk of thrombus progression or large clot burden (eg, symptomatic proximal DVT or PE), full-dose anticoagulation with platelet transfusion support to maintain counts ≥ 40,000 to 50,000/μL is advised [13,14]. In patients with lower-risk thrombotic events, such as distal DVT, isolated subsegmental PE, or catheter-related thrombosis, a 50% dose reduction or prophylactic LMWH is suggested when platelets are between 25,000 and 50,000/μL. Anticoagulation is usually temporarily held if platelet counts fall below 25,000/μL and resumed at full dose once platelets recover to >50,000/μL without the need for transfusions. In cases of active bleeding or high hemorrhagic risk, the use of intravascular filters or observation without anticoagulation may be appropriate.

To date, no randomized controlled trials (RCTs) have comprehensively addressed the optimal anticoagulation strategy for patients with CAT and thrombocytopenia. In this issue of *Research and Practice in Thrombosis and Haemostasis*, Wang et al. [15] present the protocol and rationale for the STrategies for Anticoagulation in patients with thRombocytopenia and cancer-associated Thrombosis (START) trial, a prospective, multicenter, open-label pilot RCT designed to assess the feasibility of a full-scale study investigating optimal LMWH dosing strategies in patients with CAT and thrombocytopenia. The trial will enroll patients with active cancer and acute VTE who have concurrent thrombocytopenia (platelet count < 50,000/μL), attributed to either malignancy or anticancer treatment. Study sites will include Canada and the United Kingdom. Participants will be randomized to either modified-dose LMWH adjusted by platelet counts or full-dose LMWH supported by platelet transfusions. The primary objective is feasibility, defined by the average number of patients recruited per month, with a target of 25 participants per arm. Secondary clinical outcomes include a composite of major and clinically relevant nonmajor bleeding and imaging-confirmed recurrent VTE.

START will be the first clinical trial addressing a critical knowledge gap in the management of CAT with thrombocytopenia. This multicenter international study will include all types of venous thrombosis affecting patients with solid and hematologic malignancies requiring anticoagulation. While the broad inclusion criteria enhance the generalizability (and feasibility) of the findings, heterogeneity in patient characteristics is also introduced, particularly regarding bleeding risk, and may warrant stratified analysis to allow for more accurate conclusions on dose adjustments and anticoagulation strategies. For instance, patients with hematologic malignancies often develop more severe and prolonged thrombocytopenia early due to marrow infiltration and chemotherapy, while those with solid tumors typically experience milder, later-onset, and transient thrombocytopenia from treatment. These differences highlight the need for individualized anticoagulation strategies, with hematologic cancers likely requiring more frequent dose adjustments and interruptions than solid tumors. Similarly, certain cancer subtypes may present varying bleeding risks and may need to be analyzed in subgroup analyses. For example, patients with luminal gastrointestinal or genitourinary malignancies are known to have higher bleeding tendencies, which may necessitate more tailored anticoagulation approaches. Intracranial malignancies also merit stratified evaluation due to the higher risk of major hemorrhage. Brain metastases from melanoma, renal cell carcinoma, choriocarcinoma, thyroid carcinoma, and hepatocellular carcinoma are particularly prone to hemorrhage and may benefit from more rigorous monitoring and alternative strategies, such as inferior vena cava filter placement, rather than modified doses of anticoagulation [16].

While the START trial focuses on LMWH, it will aim to refine optimal dosing strategies that have not been established through RCTs so far. However, DOACs have become the preferred treatment for VTE in the general population, including patients with cancer; their use in patients with low platelet counts is limited due to concerns of a higher bleeding risk [17]. The Apixaban Cancer Associated Thrombosis (API-CAT) trial represents a significant advancement in dosing strategies for DOACs, demonstrating that reduced-dose apixaban is noninferior to the full dose in preventing recurrent VTE, while significantly reducing the risk of clinically relevant bleeding in patients with cancer [18]. However, the study does not address dosing considerations in acute thrombosis, including in settings such as thrombocytopenia. For patients with a high bleeding risk, emerging data on factor (F)XI inhibitors offer promising alternatives, as demonstrated by phase 2 RCTs in the orthopedic setting, which show noninferior or even superior results to enoxaparin in preventing VTE, with potentially improved safety profiles [19]. To further address the unmet clinical need for anticoagulation with a lower bleeding risk, therapeutics that target the intrinsic pathway alone, such as FXI/FXIa inhibitors, are now in advanced development [20]. These novel agents have been shown to have a significantly lower bleeding risk than DOACs in head-to-head comparisons [21]. In CAT, ongoing trials are evaluating the safety and efficacy of novel FXI inhibitors, including the A multicenter, randomized, open-label, blinded endpoint evaluation, phase 3 study comparing the effect of abelacimab relative to apixaban on venous thromboembolism (VTE) recurrence and bleeding in patients with cancer associated VTE (ASTER) trial (NCT05171049), which compares abelacimab to apixaban in patients with acute CAT, and the A study comparing abelacimab to dalteparin in the treatment of gastrointestinal/genitourinary cancer and associated VTE (MAGNOLIA) trial (NCT05171075), assessing abelacimab vs dalteparin specifically in patients with gastrointestinal and genitourinary malignancies and acute VTE. However, thrombocytopenia is a key exclusion criterion in the current studies; yet these may prove to be the populations that could benefit the most if the agents prove efficacious, with the promise of reduced bleeding.

Nevertheless, this work represents an important first step (ie, a START) toward establishing a pathway for much-needed RCTs to inform anticoagulation management guidelines for CAT in the setting of thrombocytopenia. While expert panels have synthesized current recommendations based on limited observational data, the need for high-quality evidence to guide clinical decision-making in this complex scenario remains critical. Conducting interventional studies in such precarious and heterogeneous populations is undoubtedly going to be challenging. However, clinicians and patients need answers in balancing the optimal strategies for these high-risk scenarios. Researchers like Dr Wang [15] are congratulated on answering that call, and hopefully others will continue to follow.
